# Study of arsenic accumulation in rice and evaluation of protective effects of *Chorchorus olitorius* leaves against arsenic contaminated rice induced toxicities in Wistar albino rats

**DOI:** 10.1186/s40360-016-0091-8

**Published:** 2016-10-14

**Authors:** Saeed Mohammed Imran Hosen, Dipesh Das, Rupkanowar Kobi, Dil Umme Salma Chowdhury, Md Jibran Alam, Bashudev Rudra, Muhammad Abu Bakar, Saiful Islam, Zillur Rahman, Mohammad Al-Forkan

**Affiliations:** 1 Department of Genetic Engineering and Biotechnology, University of Chittagong, Chittagong, 4331 Bangladesh; 2 Phytochemistry Research Division, Bangladesh Council of Scientific and Industrial Research (BCSIR), Chittagong, Bangladesh; 3 Department of Pathology, Chittagong Medical College (CMC), Chittagong, Bangladesh

**Keywords:** Arsenic, Rice, Accumulation, Protective, *Corchorus olitorius*

## Abstract

**Background:**

In the present study, we investigated the arsenic accumulation in different parts of rice irrigated with arsenic contaminated water. Besides, we also evaluated the protective effects of *Corchorus olitorius* leaves against arsenic contaminated rice induced toxicities in animal model.

**Methods:**

A pot experiment was conducted with arsenic amended irrigation water (0.0, 25.0, 50.0 and 75.0 mg/L As) to investigate the arsenic accumulation in different parts of rice. In order to evaluate the protective effects of *Corchorus olitorius* leaves, twenty Wistar albino rats were divided into four different groups. The control group (Group-I) was supplied with normal laboratory pellets while groups II, III, and IV received normal laboratory pellets supplemented with arsenic contaminated rice, *C. olitorius* leaf powder (4 %), arsenic contaminated rice plus *C. olitorius* leaf powder (4 %) respectively. Different haematological parameters and serum indices were analyzed to evaluate the protective effects of *Corchorus olitorius* leaves against arsenic intoxication. To gather more supportive evidences of *Corchorus olitorius* potentiality against arsenic intoxication, histopathological analysis of liver, kidney, spleen and heart tissues was also performed.

**Results:**

From the pot experiment, we have found a significant (*p* ≤ 0.05) increase of arsenic accumulation in different parts of rice with the increase of arsenic concentrations in irrigation water and the trend of accumulation was found as root > straw > husk > grain. Another part of the experiment revealed that supplementation of *C. olitorius* leaves with arsenic contaminated rice significantly (*p* < 0.05) restored the altered haematological parameters and other serum indices towards the normal values. Arsenic deposition pattern on different organs and histological studies on the ultrastructural changes of liver, kidneys, spleen and heart also supported the protective roles of *Corchorus olitorius* leaves against arsenic contaminated rice induced toxicities.

**Conclusion:**

Arsenic accumulation in different parts of rice increased dose-dependently. Hence, for irrigation purpose arsenic contaminated water cannot be used. Furthermore, arsenic contaminated rice induced several toxicities in animal model, most of which could be minimized with the food supplementation of *Corchorus olitorius* leaves. Therefore, *Corchorus olitorius* can be used as a potential food supplement to the affected people of arsenic prone zone to ensure the food security.

**Electronic supplementary material:**

The online version of this article (doi:10.1186/s40360-016-0091-8) contains supplementary material, which is available to authorized users.

## Background

Arsenic (As), the king of poisons, is one of the most pivotal environmental toxicants, currently poisoning ten million people worldwide [1, 2]. Recently, As contamination of ground water is considered as the gravest natural disaster in Bangladesh. According to British Geological Survey, 51 % of ground water samples analyzed in Bangladesh (*n*= >2000) were above the WHO water standard of 0.01 mg As L-^1^ [3]. Apart from drinking purposes, ground water is widely used for crop irrigation in Bangladesh. Rice is the staple food of Bangladesh and here about 83 % of the total irrigated area is used for rice cultivation [4, 5]. Ground water irrigation-based farming practices have open up the possibility of As accumulation in rice and raised the following questions: How much As get accumulated in rice? Can As contaminated rice induce toxicities in animal? If so, what are the possible remedies? Keeping these questions in mind, this research work was carried out and according to the best of our knowledge this is the first one ever study in Bangladesh.

As is a notorious toxicant, chronic exposure of which can lead plethora of abnormalities such as skin lesions, haematological disorders, cardiovascular diseases, diabetes, neurological disorders, reproductive problems and cancer of different organs i.e., lung, skin, kidney, liver etc. [6]. As contaminated rice also have detrimental effects on human [7, 8]. Though the exact mechanism of As toxicities has not yet been fully understood, but from previous findings it is believed that As induces toxicities mostly by generating excessive reactive oxygen species (ROS). Unfortunately, still there is no specific, reliable and safe treatment for arsenicosis except some antioxidants and metal chelating agents most of which have been reported to possess toxic manifestations [9]. This increased the interest of using biologically safe medicinal plant having free radical scavenging property to counteract As induced toxicities.

Tossa jute, *Corchorus olitorius* Linn. belongs to Tiliaceae family and is a multipurpose tree widely used in Bangladeshi traditional medicine. In Bangladesh, jute leaves are consumed as popular seasonal vegetable by the local people of As contaminated area who claimed that jute leaves are capable to combat against As induced toxicities. The leaves of *C. olitorius* have been reported to possess antitumor promoters namely phytol and monogalactosyl-diacylglycerol [10]; antioxidants namely flavonoids, carotenoids and vitamin C [11–13]. Keeping the above facts in view, an experiment was designed to study the As accumulation in different parts of rice irrigated with As contaminated water and to evaluate the protective effects of jute leaves against As contaminated rice induced toxicities.

## Methods

### Pot experiment to investigate the As accumulation in rice

To investigate the As accumulation in different parts of rice, a pot experiment was conducted at University of Chittagong campus using a popular rice variety BR-28 and four treatments such as 0.0, 25.0, 50.0 and 75.0 mg/L As containing irrigation water. The experimental site had subtropical and humid climate with adequate sunshine. From the seedbed, seedlings of 35 days old were uprooted carefully in the morning and on the same day, five seedlings were transplanted on each plastic pot (having no leakage) with three replications. The seedlings which died within first week of transplantation were discarded and replaced with new seedlings. Bio-fertilizers were applied in appropriate amount to provide the necessary nutrients. Throughout the growth period, 3–4 cm water above soil level was maintained in each treatment and the irrigation was continued before 10 days of harvest. At the maturity stage, the full-grown rice plants were carefully uprooted and the rice grains were harvested. Thereafter, the collected root, straw, husk and grain samples were washed thoroughly with As-free tape water followed by several rinsing with de-ionized water to remove soil and other contaminants. After drying the washed samples in the hot air oven at 60 °C for 72 h, the samples were stored at room temperature in airtight polyethylene bags having proper labeling. Finally, the samples were digested separately according to heating block digestion procedure [14] and As concentrations were measured by Flow Injection Hydride Generation Atomic Absorption Spectrophotometer, FI-HG-AAS (iCE 3300 AA system, Thermoscientific, China) at BCSIR Laboratory, Chittagong.

### Animals and treatment

Twenty female Wistar albino rats, weighing between 160 and 170 g were collected from animal house of Jahangirnagar University, Dhaka and were allowed free access to laboratory rodent diet and water *ad-libitum* throughout the experimental period. Our institutional and national guidelines were followed for the care and use of laboratory animals throughout the experiment. For the experimental treatment, the animals were randomly divided into four groups (I, II, III and IV) containing five rats in each group. The control group (Group-I) was fed with normal laboratory pellets while groups II, III, and IV received normal laboratory pellets supplemented with arsenic contaminated rice, *C. olitorius* leaf powder (4 %) and arsenic contaminated rice plus *C. olitorius* leaf powder (4 %) respectively, for a period of 150 days.

### Preparation of *C. olitorius* leaf powder

Fresh young *C. olitorius* leaves were collected from local market and later identified by Dr. Sheikh Bokhtear Uddin, a taxonomist (Department of Botany, University of Chittagong, Bangladesh). The leaves were washed thoroughly with distilled water, sun-dried and then powdered by grinding. After that, *C. olitorius* leaf powder (4 % wt/wt) was mixed with respective pellet diet of rat as aforementioned and used throughout the experiment.

### Preparation of As contaminated rice powder

A popular rice variety of Bangladesh, BR-28, was collected from the local market and tested for background As concentration by FI-HG-AAS according to Rahman et al. [14]. No As was detected in that rice sample. After that, the rice was soaked in 200 mg/L sodium arsenite solution for 36 h and again tested for As concentration, and the amount of As accumulated in rice grain was found 46.33 ± 0.01 mg/kg. The As contaminated rice was dried, blended and mixed with the respective pellet diet and used throughout the experiment.

### Collection of blood and separation of serum

On 150^th^ day, rats were fasted overnight and sacrificed next morning by light ether anesthesia. Blood was collected through cardiac puncture. For each rat, an aliquot of blood samples was taken in a heparinated tube for haematological examination while the remaining blood sample was collected in another test tube and allowed to clot formation at room temperature for 20 min. Then, the tubes were centrifuged at 3000 r.p.m for 10 min. After centrifugation, serum samples were pipetted out & collected into pre-labeled wintrobe tubes. From collected blood and serum samples, haematological and biochemical analyses were carried out.

### Collection and preservation of different organs

After opening chest and abdomen of the rats, the liver, heart, spleen and both kidneys were carefully removed, washed in normal saline water and then immersed separately into pre-labeled 10 % formalin containing specimen container for histopathological investigations. Some portions of liver, heart, spleen and kidneys were preserved at -20 °C for detection of As.

### Haemato-biochemical assay

Using Auto-Haematology Analyzer (Beckmann, USA), different haematological indices such as total white blood cell (WBC) count, total red blood cell (RBC) count, haemoglobin (Hb) concentration and platelet count were estimated. In addition, different serum indices such as aspartate aminotransferase (AST), alanine aminotransferase (ALT), creatinine, urea, total protein, total cholesterol (TC), high density lipoprotein cholesterol (HDL-C) and triglycerides (TG) were measured by using the kits from Human Gmb H (Germany) and the analyzer (CHEM-5V3, Erba, Mannheim, Germany). Concentration of low density lipoprotein cholesterol (LDL-C) in serum was also calculated. To calculate the mean values, all the samples were analyzed in triplicate.

### Determination of As in different tissues

The concentration of As in different organs (liver, kidney, spleen, heart) was determined using FI-HG-AAS method [15]. From each organ, 0.25 g sample was weighed and taken in beaker. The samples were digested with a mixture of HClO_4_-HNO_3_ solution (ratio 1:3 v/v) at 130 °C. After removal of HNO_3_ by evaporation, the digested samples were diluted with deionized water up to 100 ml. The concentrations of As in digested samples were measured at 193.7 nm wave length and 10 mA current using Atomic Absorption Spectrophotometer equipped with As lamp. Vapour generation accessory (VGA) was used to produce hydride vapours using 0.6 % sodium borohydride and 10 Mm HCl.

### Histopathological study

At first, gross section of liver, kidney, spleen and heart tissues (preserved in 10 % formalin containing specimen container) were taken. Then the tissues were cut in longitudinal and transverse pieces, passed through ascending series of ethanol baths, cleared in toluene and embedded in paraffin. Tissues were sectioned at 5 μm and stained with Haematoxylin and Eosin (H&E). Stained sections were then mounted on glass slides with DPX and covered with a cover slip. Finally, histopathological changes were examined by light microscope and photographed using a digital camera.

### Statistical analysis

Statistical analysis was performed with SPSS for Windows V.22. All data were analyzed by using one way analysis of variance (ANOVA) followed by Duncan’s Multiple Range Test (DMART) with a p-value < 0.05 considered to be statistically significant. All the values are expressed as mean ± SEM.

## Results

In the pot experiment, we have found no plant to survive at the treatment of 75 mg/L As containing irrigation water. As accumulation in all collected samples were high for 50 mg/L and low for control (Table 1). This indicates As accumulation in different parts of rice significantly (*p* < 0.05) increased with the increase of As concentrations in irrigation water. However, the trend of As accumulation was found as root > straw > husk > grain.

**Table 1:** Accumulation of As in different parts of BR-28

As added in water (mg/L)	As in root (mg/kg)	As in straw (mg/kg)	As in husk (mg/kg)	As in grain (mg/kg)
0	4.85 ± 0.01a	1.24 ± 0.01a	0.55 ± 0.01a	0.13 ± 0.03a
25	12.39 ± 0.01b	8.63 ± 0.03b	5.90 ± 0.03b	4.83 ± 0.03b
50	45.63 ± 0.03c	36.47 ± 0.01c	12.13 ± 0.03c	11.90 ± 0.03c

Values are expressed as mean ± S.E.M. Mean in a column followed by uncommon letter differed significantly at *p* < 0.05

Results of the haematological profile of both control and treated groups are depicted in Table 2. In Group-II, we noticed significantly (*p* < 0.05) decreased concentration of Hb, total WBC count and total RBC count with a non-significant decreased concentration of platelet count when compared with the control (Group-I). Supplementation of *C. olitorius* with As contaminated rice (Group-IV) significantly (*p* < 0.05) attenuated the As induced haematological alterations. Administration of *C. olitorius* with normal diet (Group-III) showed no alterations in these parameters and did not differ significantly from Group-I.

**Table 2:** Effect of *C. olitorius* on haematological parameters of albino rats

Groups	Hb gm/dl	WBC /cmm	RBC x 10^6^/cmm	Platelets x 10^3^/cmm
Group-I (Control)	13.8 ± 0.12	6300 ± 122.50	5.16 ± 0.10	180 ± 3.20
Group-II (As cont. rice)	* 13.3 ± 0.20	* 4860 ± 97.98	* 4.68 ± 0.16	NS 170 ± 5.50
Group-III (Leaf)	NS 13.82 ± 0.10	NS 6360 ± 435.43	NS 5.14 ± 0.10	NS 176 ± 2.45
Group-IV (As cont. rice + Leaf)	** 13.76 ± 0.11	** 6200 ± 339.12	** 5.02 ± 0.10	NS 174 ± 4.00

Values are expressed as mean ± S.E.M. NS denotes non-significant; *denotes significantly different from control at *p* < 0.05; **denotes significantly different from the arsenic-treated group at *p* < 0.05

**Fig. 1:**
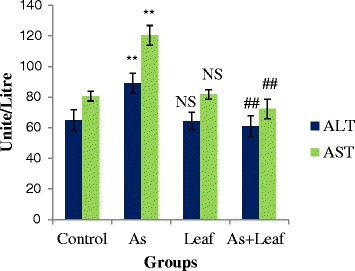
Effects of *C. olitorius* leaves on liver marker enzymes. Each bar represents the mean ± S.E.M. NS denotes non-significant; **denotes significantly different from control at *p* < 0.05; ## denotes significantly different from the arsenic-treated group at *p* < 0.05

In order to assess the extent of hepatic damage, activities of two liver marker enzymes—AST and ALT were estimated. We observed that the activities of serum ALT and AST enzymes were significantly (*P* < 0.05) elevated in Group-II when compared with the Group-I. However, administration of *C. olitorius* with normal diet (Group-III) did not show any significant (*P* < 0.05) effect on liver marker enzymes. A significant (*P* < 0.05) lower levels of ALT and AST enzymatic activities were noticed in As contaminated rice treated rats supplemented with *C. olitorius* leaves (Group-IV) as shown in Fig. 1.

**Fig. 2:**
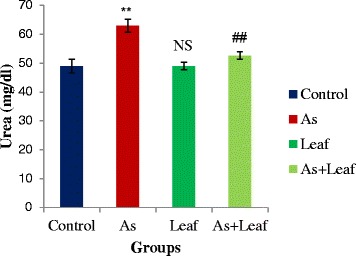
Effects of *C. olitorius* leaves on serum urea levels. Each bar represents the mean ± S.E.M. NS denotes non-significant; **denotes significantly different from control at *p* < 0.05; ## denotes significantly different from the arsenic-treated group at *p* < 0.05

Figure 2 exhibits the serum urea levels of control and experimental rats. Elevated levels of serum urea is a hallmark of renal dysfunction caused by As. In this study, we have found significant (*p* < 0.05) elevation of serum urea levels in As contaminated rice intoxicated rats (Group-II) when compared with control rats (Group-I). Food supplementation of *C. olitorius* leaves (Group-IV) significantly (*p* < 0.05) reduced the elevated levels of serum urea compared to As-treated rats.

**Fig. 3:**
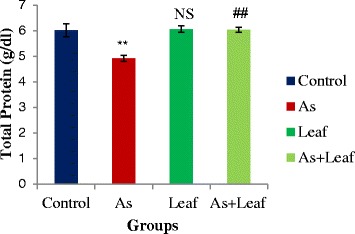
Effects of *C. olitorius* leaves on serum total protein. Each bar represents the mean ± S.E.M. NS denotes non-significant; **denotes significantly different from control at *p* < 0.05; ## denotes significantly different from the arsenic-treated group at *p* < 0.05

As shown in Fig. 3, we observed decreased serum protein in As intoxicated rats (Group-II) when compared with control rats (Group-I). Intriguingly, we noticed that supplementation of *C. olitorius* leaves with As contaminated rice (Group-IV) significantly (*p* < 0.05) increased the serum protein level. However, there was no significant difference in serum protein level between the control and *C. olitorius* leaves groups (Group-I and Group-III).

Table 3 depicts the results of *C. olitorius* effects on serum lipid profiles of control and experimental animals. In the present study, we determined the levels of serum TG, TC, HDL-C and LDL-C in four groups of albino rats. When compared with control, we observed that serum TG, TC and LDL-C levels were significantly (*p* < 0.05) increased in As contaminated rice treated rats (Group-II). In addition, HDL-C level was significantly (*p* < 0.05) reduced in Group-II compared to Group-I. Remarkably, these altered serum lipid profiles significantly (*p* < 0.05) restored to normal values when rats were fed with As contaminated rice plus *C. olitorius* leaves (Group-IV). However, co-administration of *C. olitorius* with normal diet (Group-III) did not show any alteration in serum lipid profiles and did not differ significantly from Group-I.

**Table 3:** Effect of *C. olitorius* on serum lipid profile of albino rats

Groups	Total Cholesterol mg/dl	HDL mg/dl	LDL mg/dl	TG mg/dl
Group-I (Control)	79.22 ± 1.52	32.33 ± 1.47	30.41 ± 2.58	82.91 ± 0.96
Group-II (As cont. rice)	* 88.35 ± 1.61	* 27.4 ± 1.89	* 43.23 ± 1.61	* 88.6 ± 1.81
Group-III (Leaf)	NS 78.42 ± 1.10	NS 32.63 ± 0.99	NS 29.37 ± 1.82	NS 82.11 ± 1.08
Group-IV (As cont. rice + Leaf)	** 78.23 ± 0.94	** 32.63 ± 1.27	** 28.90 ± 2.00	** 83.52 ± 1.86

Values are expressed as mean ± S.E.M. NS denotes non-significant; *denotes significantly different from control at *p* < 0.05; **denotes significantly different from the arsenic-treated group at *p* < 0.05

**Fig. 4:**
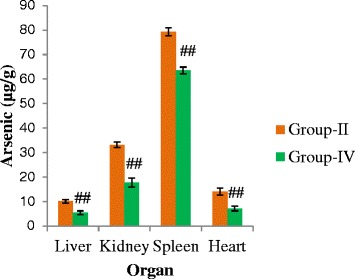
Effects of *C. olitorius* leaves on As deposition pattern. Each bar represents the mean ± S.E.M. NS denotes non-significant; **denotes significantly different from control at *p* < 0.05; ## denotes significantly different from the arsenic-treated group at *p* < 0.05

Figure 4 shows the effect of *C. olitorius* leaves on As deposition pattern in different organs of rat. From the FI-HG-AAS analysis of the organ samples, we found significantly (*p < 0.05*) lower amount of As deposition in Group-IV than Group-II. In addition, we also noticed in both groups that higher amount of As was deposited in spleen than other organs and the trend of As accumulation was as spleen > kidney > heart > liver.

**Fig. 5:**
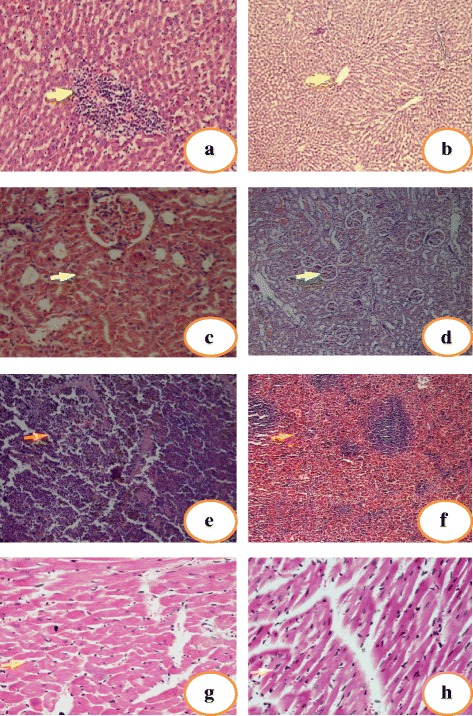
Liver (**a**, **b**), kidney (**c**, **d**), spleen (**e**, **f**) and heart (**g**, **h**) sections from Group-II (*left side*) and Group-IV (*right side*) respectively. **a** depicts the necrosis in liver section of Group-II. **b** depicts the normal liver architecture of Group-IV. **c** depicts the tubular epithelium necrosis in kidney section of Group-II. **d** depicts the normal kidney architecture of Group-IV. **e** depicts the diffusingly enlarged white pulp in spleen section of Group-II. **f** depicts slightly enlarged white pulp in spleen section of Group-IV. **g** depicts mild cellular edema in heart section of Group-II. **h** depicts normal cardiac architecture of Group-IV

In order to gather more supportive evidences regarding the protective behavior of *C. olitorius* leaves against As contaminated rice induced toxicities, histopathological assessments were undertaken. Control rats (Group-I) and *C. olitorius* leaves treated rats (Group-III) showed a normal architecture of the liver whereas exposure to As contaminated rice (Group-II) resulted various pathological changes in liver architecture as characterized by mild to marked venous congestion, sinusoidal dilation, multiple foci of mononuclear cell infiltration, focal haemorrhages, varied degree of necrosis and degenerative changes in the hepatocytes (Fig. 5a). However, As contaminated rice with *C. olitorius* supplementation (Group-IV) showed almost normal liver architecture (Fig. 5b). Light microscopic observations on kidney sections of Group-II revealed glomerulonephritis, proximal tubular necrosis, epithelial damage and loss of nuclei (Fig. 5c). Co-administration of *C. olitorius* with As contaminated rice reduced such changes and kept the kidney architecture similar to that of normal (Fig. 5d). From the spleen section of Group-II, increased number of apoptotic cells, necrotic cells and macrophages were observed with diffusingly enlarged white pulp which indicate the disturbances of functional activity of spleen (Fig. 5e), whereas in Group-IV, these perturbations were not pronounced (Fig. 5f). The effect of As contaminated rice on heart was not pronounced as compared to other organs. The cardiac histology of Group-II revealed mild cellular edema and lukocytic infiltration (Fig. 5g) while rest of the groups showed normal cardiac architecture (Fig. 5h).

## Discussion

The current research work was designed to investigate the As accumulation in different parts of rice and to evaluate the protective effects of *C. olitorius* leaves against As contaminated rice induced toxicities. To accomplish the first objective, a pot experiment was conducted where accumulation of As were found in high amounts in root followed by straw, husk and grain. In addition, the highest amount of As accumulation in different parts of rice was found in 50 mg/L As containing irrigation water which suggests that for irrigation purpose, As contaminated water cannot be used. Moreover, we noticed that As accumulation in different parts of rice were significantly higher than the permissible limits of WHO. Therefore, As contaminated rice and straw should not be consumed as human feed and cattle feed respectively. In the absolute control condition (0 mg/L), a trace amount of As accumulation was found that might be due to the background As in the soil. These findings are affirmative with the findings of Abedin et al. [16] and Imamul Huq et al. [17].

It is noticed from the earlier investigations that As can exhibit its toxicities by inducing haematological perturbations. Similar findings were also observed in this study. In the present study, we noticed significant reduction of Hb, total RBC count and total WBC count in As contaminated rice treated group (Group-II). The reduced Hb concentration and total RBC count might be due to binding ability of As to Hb that leads to inhibition of heme synthesis pathway [18]. Furthermore, decreased level of total WBC count might be due to apoptotic effect of As on plasma cells as also studied by Rousselot et al. [19]. We found no significant differences in platelet count among the groups in this study. Similar findings were also observed by Ferzand et al. [20]. A significant improvement of altered haematological parameters was observed in ameliorative group (Group-IV) compared to Group-II that confirms the beneficial roles of *C. olitorius* in restoring hematological parameters.

Arsenic can generate free radicals that promote lipid peroxidation by attacking polyunsaturated fatty acids in membranes, setting off a free radical chain reaction sequence. Peroxidation of lipid is known to cause membrane disruption, resulting in the loss of membrane integrity and leakage of microsomal enzymes [21]. AST and ALT are two prominent liver marker enzymes which are cytoplasmic in origin and are released into circulation after cellular damage [22]. That’s why increased activities of these enzymes in serum are an indication of hepatic damage. In the present study, we observed that As contaminated rice administration substantially increased the serum AST and ALT activities as also reported by Islam et al. [23]. On the contrary, co-administration of *C. olitorius* leaves as a food supplement remarkably reduced the As induced elevation of serum AST and ALT activities. These findings suggest the hepatoprotective role of *C. olitorius* leaves against As contaminated rice induced liver injury in rat.

Renal dysfunction is one of the major health effects of chronic As exposure, and increased levels of serum urea have been reported to be associated with renal dysfunction [24]. In the present study, we have also found increased serum urea levels in As contaminated rice intoxicated rats which is an indication of the detrimental effects of As on kidney. *C. olitorius* leaves potentially reduced the As induced elevation of serum urea levels which indicates its nephroprotective role.

Reduced protein synthesis is associated with As intoxication as showed by Mehta and Hundal [25] and our results are corroborating with their findings. We observed reduced serum protein level in Group-II compared to Group-I. This reduction might be attributed to increased proteolytic activity or reduced protein synthesis or destruction of hepatic protein synthesizing sub-cellular structures. In addition, it is also possible that severe nephrotoxic lesions caused drainage of protein through the urine, resulting in hypoproteinaemia. Interestingly, co-administration of *C. olitorius* leaves with As contaminated rice significantly (*p* < 0.05) reversed the serum protein level towards control. One possibility for increasing serum protein level might be due to antioxidant activity of *C. olitorius* leaves with a significant amount of α-tocopherol equivalent Vitamin E [26] where vitamin E could decrease hepatic insulin resistance allowing insulin to stimulate the incorporation of amino acids into protein [27].

Cardiovascular disease is one of the prominent causes of As related mortality [28]. Increased levels of TG, TC and LDL-C are often associated with cardiovascular diseases such as atherosclerosis. In this study, we also observed elevated levels of TG, TC and LDL-C in As contaminated rice treated rats (Group-II) which were reversed to normal levels with the supplementation of *C. olitorius* leaves (Group-IV). This may be an indication of the anti-hyper lipidemic property of *C. olitorius* leaves as also reported by Adedosu et al. [29]. These findings open up the possibility to employ the *C. olitorius* leaves in treating cardiovascular diseases in near future.

Due to chronic exposure, As is known to accumulate in different organs such as spleen, kidney, heart, liver etc. Among the organs, spleen is more prone to accumulate As than other organs as studied by Nasir et al. [30] and our results are in full agreement with their findings. Lower As deposition was found in organs of rat treated with As contaminated rice plus *C. olitorius* than those treated with As contaminated rice alone. *C. olitorius* leaves might have thiol-containing amino acids which could play role to reduce tissue As burden in rat.

Histopathological evaluation of different organs revealed that As contaminated rice caused various pathological alterations in the tissues. However, supplementation of *C. olitorius* leaves with As contaminated rice could prevent the pathological alterations and also maintain the normal histo-architecture of tissues.

## Conclusion

Conclusively, this investigation has revealed that As contaminated irrigation water based farming practices lead to As accumulation in different parts of rice and in all cases As accumulation exceed the permissible limits of WHO. Therefore, As contaminated water should not be used for irrigation purposes. Furthermore, As contaminated rice can induce several toxicities in animal model. Notably, most of the toxicities can be minimized through supplementation of *C. olitorius* leaves. Therefore, *C. olitorius* can be supplemented with the normal diet to the affected people of As prone zone. However, the exact mechanism of *C. olitorius* in neutralizing As-induced toxicities in vivo is still obscure. Therefore, further molecular and biochemical investigations are needed to explore the mode of its action before use as a potential candidate to remediate As contaminated rice induced toxicities.

## Additional file

Additional file 1: Supplementary data for arsenic accumulation and haemato biochemical parameter analysis in rat model. (XLSX 60 kb)

## Abbreviations

ALT: Alanine aminotransferase
As: Arsenic
AST: Aspartate aminotransferase
Hb: Haemoglobin
HDL-C: High density lipoprotein cholesterol
LDL-C: Low density lipoprotein cholesterol
RBC: Red blood cell
TC: Total cholesterol
TG: Triglycerides
WBC: White blood cell

## References

[1] Hughes MF, Beck BD, Chen Y, Lewis AS, Thomas DJ (2011). Arsenic exposure and toxicology: a historical perspective. Toxicol Sci.

[2] Smeester L, Rager JE, Bailey KA, Guan X, Smith N, García-Vargas G (2011). Epigenetic changes in individuals with arsenicosis. Chem Res Toxicol.

[3] British Geological Survey. Groundwater studies for arsenic contamination in Bangladesh: phase 1. 2000. http://bicn.com/acic/resources/infobank/bgs-mmi/risumm.htm. Accessed Sept 2007.

[4] Dey MM, Miah MNI, Mustafi BAA, Hossain M, Evenson RE, Herdt RW, Hossain M (1996). Rice production constraints in Bangladesh: Implications for further research priorities. Rice research in Asia: progress and priorities.

[5] Abedin MJ, Feldmann J, Meharg AA (2002). Uptake kinetics of arsenic species in rice plants. Plant Physiol.

[6] Kapaj S, Peterson H, Liber K, Bhattacharya P (2006). Human health effects from chronic arsenic poisoning–a review. J Environ Sci Health A.

[7] Martınez M, Pan J, Polya DA, Giri AK. High arsenic in rice is associated with elevated genotoxic effects in humans. 2013. Scientific reports, 3 10.1038/srep02195 PMC6505394 23873074

[8] Melkonian S, Argos M, Hall MN, Chen Y, Parvez F, Pierce B, Cao H, Aschebrook-Kilfoy B, Ahmed A, Islam T, Slavcovich V, Gamble M, Haris PI, Graziano JH, Ahsan H (2013). Urinary and dietary analysis of 18,470 Bangladeshis reveal a correlation of rice consumption with arsenic exposure and toxicity. PLoS One.

[9] Shi H, Shi X, Liu KJ (2004). Oxidative mechanism of arsenic toxicity and carcinogenesis. Mol Cell Biochem.

[10] Furumoto T, Wang R, Okazaki K, Hasan AFMF, ALI MI, Kondo A, Fukui H (2002). Antitumor promoters in leaves of jute (Corchorus capsularis and Corchorus olitorius). Food Sci Technol Res.

[11] Khan MSY, Bano S, Javed K, Mueed MA (2006). A comprehensive review on the chemistry and pharmacology of Corchorus species – a source of cardiac glycosides, triterpenoids, ionones, flavonoids, coumarines steroids and some other compounds. J Sci Ind Res.

[12] Zeid AH (2002). Stress metabolites from Corchorus olitorius L. leaves in response to certain stress agents. Food Chem.

[13] Azuma K, Nakayama M, Koshioka M, Ippoushi K, Yamaguchi Y, Kohata K, Yamauchi Y, Ito H, Higashio H (1999). Phenolic antioxidants from the leaves of Corchorus olitorius L. J Agric Food Chem.

[14] Rahman MA, Hasegawa H, Rahman MM, Rahman MA, Miah MA (2007). Accumulation of arsenic in tissues of rice plant (Oryza sativa L.) and its distribution in fractions of rice grain. Chemosphere.

[15] Hirano A (1994). Hirano bodies and related neuronal inclusions. Neuropathol Appl Neurobiol.

[16] Abedin MJ, Cotter-Howells J, Meharg AA (2002). Arsenic uptake and accumulation in rice (Oryza sativa L.) irrigated with contaminated water. Plant Soil.

[17] Imamul Huq SM, Sultana S, Chakraborty G, Chowdhury MT (2011). A mitigation approach to alleviate arsenic accumulation in rice through balanced fertilization. Appl Environ Sci.

[18] Gupta R, Flora SJ (2006). Protective effects of fruit extracts of Hippophae rhamnoides L. against arsenic toxicity in Swiss albino mice. Hum Exp Toxicol.

[19] Rousselot P, Larghero J, Labaume S, Poupon J, Chopin M, Dosquet C, Marolleau JP, Janin A, Brouet JC, Fermand JP (2004). Arsenic trioxide is effective in the treatment of multiple myeloma in SCID mice. Eur J Haematol.

[20] Ferzand R, Gadahi JA, Saleha S, Ali Q (2008). Histological and haematological disturbance caused by arsenic toxicity in mice model. Pak J BiolSci.

[21] Tarantino G, Di Minno MN, Capone D (2009). Drug-induced liver injury: is it somehow foreseeable?. World J Gastroenterol.

[22] Lin SC, Chung TC, Lin CC, Ueng TH, Lin YH, Lin SY, Wang LY (2000). Hepatoprotective effects of Arctium lappa on carbon tetrachloride-and acetaminophen-induced liver damage. Am J Chin Med.

[23] Islam K, Haque A, Karim R, Fajol A, Hossain E, Salam KA, Ali N, Saud ZA, Rahman M, Rahman M, Karim R (2011). Dose-response relationship between arsenic exposure and the serum enzymes for liver function tests in the individuals exposed to arsenic: a cross sectional study in Bangladesh. Environ Health.

[24] Wang JP, Wang SL, Lin Q, Zhang L, Huang D, Ng JC (2009). Association of arsenic and kidney dysfunction in people with diabetes and validation of its effects in rats. Environ Int.

[25] Mehta M, Hundal SS (2013). Induction of oxidative stress by sub-acute oral exposure of sodium arsenite in female rats. Indian J Appl Res.

[26] Islam MM (2013). Biochemistry, medicinal and food values of jute (Corchorus capsularis L. and C. olitorius L.) leaf: A review. Int J Enhanc Res Sci Technol Eng.

[27] Manning PJ, Sutherland WH, Walker RJ, Williams SM, De Jong SA, Ryalls AR, Berry EA (2004). Effect of high-dose vitamin E on insulin resistance and associated parameters in overweight subjects. Diabetes Care.

[28] Chen CJ, Chiou HY, Chiang MH, Lin LJ, Tai TY (1996). Dose-response relationship between ischemic heart disease mortality and long-term arsenic exposure. Arterioscl Throm Vas.

[29] Adedosu OT, Akanni OE, Afolabi OK, Adedeji AL (2015). Effects of Corchorus olitorius extract on certain antioxidants and biochemical indices in sodium arsenite exposed rats. Am J Phytomed & Clin Therap.

[30] Nasir M, Misbahuddin M, Ali SK (2004). Selenomethionine: A therapeutic adjunct to arsenic-free water in reducing tissue arsenic load. J Med Sci Res.

